# Interbirth intervals: Intrafamilial, intragenomic and intrasomatic conflict

**DOI:** 10.1093/emph/eou002

**Published:** 2014-02-07

**Authors:** David Haig

**Affiliations:** Department of Organismic and Evolutionary Biology, Harvard University, Cambridge, MA, USA

**Keywords:** parent–offspring conflict, interbirth interval, genomic imprinting, microchimerism, secondary infertility

## Abstract

**Background and objectives**: Interbirth intervals (IBIs) mediate a trade-off between child number and child survival. Life history theory predicts that the evolutionarily optimal IBI differs for different individuals whose fitness is affected by how closely a mother spaces her children. The objective of the article is to clarify these conflicts and explore their implications for public health.

**Methodology**: Simple models of inclusive fitness and kin conflict address the evolution of human birth-spacing.

**Results**: Genes of infants generally favor longer intervals than genes of mothers, and infant genes of paternal origin generally favor longer IBIs than genes of maternal origin.

**Conclusions and implications**: The colonization of maternal bodies by offspring cells (fetal microchimerism) raises the possibility that cells of older offspring could extend IBIs by interfering with the implantation of subsequent embryos.

## Abstract (precis)

A longer time between births increases child survival. Night-waking to suckle may benefit infants by delaying a mother's return to fertility. Cells of fetal origin colonize the mother's body during pregnancy and persist until subsequent pregnancies. These cells will have been selected to extend inter-birth intervals.

## BACKGROUND AND OBJECTIVES

The time between the birth of one child and the birth of the next shapes family structures and has implications for public health. Short interbirth intervals (IBIs) are associated with increased risk of death in childhood, both for the child whose birth begins the interval and for the child whose birth ends it [1–3]. Long IBIs, when these are not planned or the result of sexual abstinence, may provide evidence of underlying infertility [4].

IBI is a key variable in life-history theory [5]. Mothers on limited budgets face an evolutionary trade-off between investing less in each of a larger number of offspring or more in each of a smaller number [6]. This trade-off implies evolutionary conflict between genes of mothers and offspring because maternal fitness will have been maximized by less investment per child than maximizes each child’s fitness [7]. Offspring fitness will have been maximized by longer IBIs than optimal for maternal fitness [8, 9].

Genetic boundaries within families are less clear cut than was once thought. Cells move in both directions across the placenta, from mother to fetus and from fetus to mother. These self-transplanted cells can maintain themselves for decades in their new locations [10]. This phenomenon has attracted recent medical interest but has been largely ignored by evolutionary biologists, even though ubiquitous microchimerism suggests unexplored possibilities of mother–offspring conflict and sibling rivalry within ‘individual’ bodies.

This article has two major parts. The first presents simple heuristic models of evolutionary conflict over optimal IBIs with the intent of clarifying factors that should be considered in understanding the evolution of human birth-spacing. The second considers the implications, for IBIs and maternal fertility, of the colonization of the mother’s body by cells from each of her offspring.

## MODELS

### Spousal and affinal conflict

The models of this article apply to all sexual organisms that produce offspring one at a time, but I will employ the terminology of husbands and wives because I need a simple way to distinguish between mother’s partners who may be the father of a child and the genetic father of the child. I reserve the adjectives maternal and paternal for genes in mothers and fathers and use the adjectives madernal and padernal for genes ‘derived from’ mothers and fathers (here I revert to adjectives first used in [11] in place of the universally unloved madumnal and padumnal).

A mother’s inclusive fitness (*W*_M_) can be represented as a sum of contributions from her current infant (*V*), from other offspring (*R*) and from other matrilineal kin (*S*_M_). *V* is assumed to be an increasing function of time (*t*) from the infant’s birth until next birth. *R* is assumed to be a decreasing function of *t* in the neighborhood of optimal trade-offs. *S*_M_ increases with *t* if longer IBIs reduce competition for resources among matrilineal kin. The marginal effect of change in *t* is
(1)
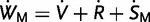

using dots to denote derivatives with respect to *t*.

Effects of *t* on the inclusive fitness of the mother’s husband (*W*_H_) need to account of the possibilities that he is not the infant’s father and that he is not the father of the mother’s future offspring. If the husband has probability *p* of being the current child’s father and expects a proportion *q* of the mother’s future offspring, then
(2)
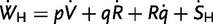



The four right-hand terms represent marginal effects of longer IBIs: the first is a positive contribution from increased fitness of the infant, the second a negative contribution from a decrease in the mother’s residual reproductive value, the third a negative contribution from the increased probability that some other male will father the mother’s future offspring (assuming *q* to be a decreasing function of *t*), the fourth summarizes ‘social’ effects via the husband’s kin, including effects on his production of offspring with other mothers.

Subtracting (1) from (2) identifies spousal divergence of genetic interests
(3)
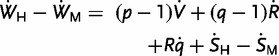



If *p* = *q* = 1 (strict life-time monandry), the first three right-hand terms are zero, and conflict between spouses arises solely from effects on their respective kin. Husbands favor shorter IBIs than wives if 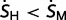 but longer IBIs if 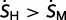. Lower fertility of a marriage is favored by whichever spouse is more closely related to other individuals who experience increased competition from an additional child.

At the wife’s optimal IBI, 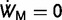 and 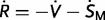. Therefore,
(4)
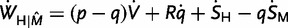



Equation (4) describes the marginal effect on the husband’s inclusive fitness of changes in *t* at the wife’s optimal IBI. The first term on the right has the same sign as (*p* − *q*). A husband favors delay of his wife’s next birth if his relatedness to her current offspring is greater than his relatedness to her future offspring but earlier return to fertility if the reverse. The second term is negative and favors earlier return to fertility by his wife when his expected share of her residual reproductive value falls with longer delays. Conceptions of additional offspring become time-sensitive opportunities for a husband who risks replacement by another male. The third and fourth terms represent social effects via husband’s and wife’s kin.

### Intragenomic and intergenerational conflict

The effect of changes of IBI for the inclusive fitness of unimprinted genes in offspring can be obtained by averaging the separate effects for madernal and padernal inclusive fitness
(5)
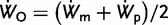

(6)
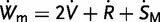

(7)
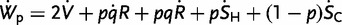



where 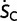 represents effects on cuckolders’ kin. Equation (7) was derived under the simplifying assumptions that husbands and cuckolders are unrelated and that children conceived by cuckoldry do not have full-sibs.


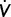
 is given double weight in (6) compared to (1) because a madernal allele is definitely present in the infant but a maternal allele has only one chance in two of being present. Therefore,
(8)
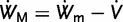



Madernal alleles favor longer IBIs than maternal alleles because 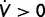.

Subtraction of (5) from (8) defines the potential for mother–offspring conflict.
(9)
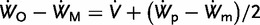



Conflict intensifies as infant fitness is more sensitive to changes in IBI and as padernal alleles gain greater benefits than madernal alleles from prolongation of the IBI.

Equation (9) simplifies to
(10)
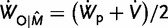

when evaluated at 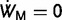. The necessary condition for (10) to be negative is
(11)
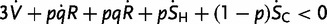



I can think of no plausible scenario in which (11) would be satisfied. Therefore, I conclude that (10) is positive and unimprinted genes of offspring favor longer IBIs than maternal genes. This conclusion is reinforced by consideration of the known effects of imprinted genes on suckling and night waking by infants (discussed below).

Subtraction of (6) from (7) yields the potential for madernal–padernal conflict
(12)
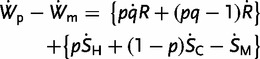



The factors responsible for intragenomic conflict are grouped into two ‘mating’ terms (left-hand brackets) and three ‘social’ terms (right-hand brackets). The mating terms pull the optimal value of *t* for padernal alleles in opposite directions. The first mating term is negative and favors shorter intervals. It represents effects of reduced padernal relatedness to subsequent sibs arising from time-dependent changes in who fathers a woman’s children. The second mating term is positive and favors longer intervals. It represents the reduction in *R* due to longer IBIs weighted by (*pq* − 1), the difference of padernal and madernal ‘interest’ in the mother’s other offspring. The three social terms represent the difference between effects on genetic fathers’ kin (sum of the first two terms) and effects on the mothers’ kin (third term).

Prader–Willi syndrome (PWS) and Angelman syndrome (AS) are caused by deletion of a cluster of imprinted genes on chromosome 15 [12]. Infants without a paternally derived cluster are diagnosed with PWS and exhibit profound deficits in suckling and rarely wake to feed [13]. In contrast, infants without a maternally derived cluster are diagnosed with AS and wake frequently at night [14]. These phenotypes suggest night waking and suckling are arenas of conflict within infant genomes with genes of paternal origin favoring more intense suckling and more fragmented sleep [15–17].

Blurton Jones and da Costa proposed that night waking to suckle is an adaptation of infants and toddlers to suppress ovarian function in their mothers, thereby extending the IBI and delaying the birth of a competitor for maternal care [8]. The phenotypes of PWS and AS suggest that padernal genes of infants have been selected to favor longer subsequent IBIs than madernal genes of infants. This implies that Equation (12) was positive during recent human evolution and, as a further implication, that unimprinted genes of infants favor longer IBIs than maternal genes.

## COLLECTIVE BODIES

### Microchimerism

Maternal bodies are engrafted by fetal cells during pregnancy, and fetal bodies are engrafted by maternal cells. The engrafted cell lineages may persist for decades [10]. Thus, a multiparous mother can contain within her body cells derived from each of her offspring and from her own mother. Bidirectional movement of cells across the placenta raises the possibility of secondary engraftment. Fetuses could be colonized by cells of older sibs present in their mother’s body or by cells of their maternal grandmother. The colonization of mothers’ bodies by offspring cells is usually inferred from the presence of cells derived from sons, detected by their Y chromosome, because of the technical difficulties of detecting cells derived from daughters [18].

From an evolutionary perspective, engrafted cells are extensions of the genetic individual of whom the cells are disjunct fragments and are predicted to evolve effects that increase that individual’s inclusive fitness [19]. I previously suggested that engrafted fetal cells could manipulate the mother’s body for offspring benefit [20] and now develop that idea further. In particular, the arguments of previous sections suggest that fetal cells could be selected to promote prolongation of subsequent IBIs.

Offspring usually benefit from their mothers’ continued well-being. Therefore, natural selection will have opposed major costs of engrafted cells to a mother’s health and favored benefits to mothers. Such benefits have been reported [21, 22]. However, offspring are more closely related to themselves than to sibs, whereas mothers are equally related to all offspring. Therefore, if it were possible, offspring cells in mother’s bodies should favor their own child at the expense of its sibs. Engrafted cells could have many effects during pregnancy that benefit the fetus from which they were derived but their postnatal ability to discriminate in favor of their own child will be limited.

Microchimerism internalizes the family within maternal bodies. Offspring cells in maternal blood do not increase in abundance with parity and the cells of a mother’s mother become less abundant as women have more children. These observations suggest that the different populations of engrafted cells may compete for a limited niche within their shared host [23]. Circulating cells of the mother’s mother increase in abundance from the first to third trimester of pregnancy [24], prompting the question what such cells could do during pregnancy to enhance the inclusive fitness of the mother’s mother (the fetus’s maternal grandmother).

### Postnatal favoritism

There are two obvious routes by which engrafted cells could direct postnatal benefits toward their own child. The first is via effects on mammary glands to enhance milk production. The second is by delay of the birth of a younger sib, either by contraceptive effects on the mother’s brain or ovary or by targeting embryos after conception in the fallopian tube or uterus.

Male cells are commonly detected in human breast tissue [25] but less often in women with breast cancer [26]. Male cells are also less frequent in the peripheral blood of women who subsequently develop breast cancer than in those who remain cancer-free [27]. The nature of these associations between fetal microchimerism and breast cancer is unknown, but I offer the following conjecture: fetal cells have evolved to promote differentiation of mammary epithelial cells during pregnancy to enhance milk delivery to infants after birth. Therefore, in the absence of microchimerism, the breast contains a larger population of cancer susceptible, undifferentiated cells. Such a mechanism could help explain why pregnancy is protective against the development of breast cancer [28].

Microchimerism is also common in endometrial samples from parous women [29]. In this location, the detached representatives of older offspring would be ideally placed for interfering with the implantation or growth of subsequent embryos. Fetally derived cells in mothers’ bodies share many features with hematopoietic stem cells that differentiate and contribute to endometrial structures in transplant recipients [30]. The interesting question arises whether fetal cells ever contribute to endometrial structures in their mother, including at extrauterine sites (endometriosis).

Infants will have benefited from prolongation of maternal postpartum infertility. A direct way to achieve this end would be for engrafted cells to impede the successful establishment of pregnancy by one or more subsequent embryos. Indeed, male cells in peripheral blood are more readily detected in women who have experienced a pregnancy loss than in otherwise matched women who have not experienced a loss [31]. If embryo losses are non-discriminatory, in the sense that subsequent embryos are targeted independently of their genotype, then the principal benefit to genes of the infant would be delay of the mother’s next birth. The benefits of further delay should diminish with time relative to the kin-selected benefits of an extra sib.

More severe effects on fertility are possible if haplotypes in engrafted cells discriminate against particular embryonic genotypes either directly or via effects on the mother’s immune system. Consider a *D* haplotype expressed in maternally engrafted cells of a *Dd* fetus that causes the abortion of subsequent *dd* embryos. The elimination of *dd* embryos benefits the *D* haplotype both by prolonging the IBI for an existing *D*-bearing infant and by reducing the time from the loss of a *dd* embryo until the next implantation of a *D*-bearing embryo. In this scenario, liveborn children exhibit a deficiency of *dd* homozygotes and segregation distortion in favor of *D* at the expense of *d*. The scenario resembles models of gestational drive [32, 33] except that the driving *D* haplotype is expressed in offspring rather than mothers.

Secondary recurrent miscarriage (SRM) is defined as three or more consecutive miscarriages after a live birth [34]. Suppose one parent is *Dd* and the other *dd*, and that all *dd* embryos abort after engraftment of cells from the first *Dd* offspring. Given the first loss of a *dd* embryo, the probability that the next two pregnancies will also be *dd* and aborted is one quarter, satisfying the definition of SRM. The *D* allele can benefit despite a substantial reduction in maternal fertility because it is absent from aborted embryos but present in their replacements. A geneticist evaluating such a system runs the risk of blaming the victim and excusing the aggressor by identifying the *D* allele as ‘protective’ and the *d* allele as ‘predisposing’ [32].

### Battling brothers

In a study of 358 women with unexplained SRM, the sex ratio of children born prior to three or more consecutive miscarriages was 1.49, but 0.76 for children born after the miscarriages [34]. These ratios suggest that male births are more likely to be followed by multiple miscarriages and that male fetuses preferentially miscarry. Thus, earlier-born males appear to have stronger negative effects on subsequent sibs, especially on brothers, than earlier-born females. This pattern is compatible with a scenario in which competition among brothers has been more intense than competition among sisters, because older brothers would then have had stronger evolutionary incentives for impairing the competitive ability of younger brothers.

Early-born males are statistically associated with additional fitness costs for later-born sibs, especially brothers. Younger sibs of older brothers have lower birth weights than younger sibs of older sisters, with the reduction greater for younger brothers than for younger sisters [35]. Older brothers, but not sisters, increase the probability that a male will have homosexual orientation whether or not the brothers grow up together [36]. These effects are usually ascribed to immunization of the mother by male-specific antigens during pregnancy with immune-mediated effects on subsequent sons [37]. However, effects mediated via engrafted cells of older brothers in mother’s bodies should also be considered (the mechanism could still involve male-specific antigens).

## CONCLUSIONS AND IMPLICATIONS

The models of this article formalize hypotheses that offspring have evolved to favor longer IBIs than mothers and that padernal genes of infants have evolved to favor longer IBIs than madernal genes [8, 9, 17]. The latter hypothesis has been misunderstood by colleagues to be a claim that husbands favor longer IBIs than their wives (a claim that has been vehemently disputed). One purpose of this article has been to clarify the difference. Comparison of Equations (3) and (12) clearly shows that the two claims are distinct with respect to inclusive fitness, in large part, because males can never be certain that any particular offspring is their own.

Women often conceive sooner after the birth of a child than they desire. If women’s return to fertility has been shaped by natural selection, then the mismatch between a woman’s preference and the fertility of her body suggests her desired IBI is longer than the IBI that maximized women’s fitness. In evolutionary arguments about human life history, there is a risk of confusing two concepts of preference. The first is what human individuals desire, the proper concern of medicine and public health, and the second is what is ‘favored’ by natural selection, the subject of this paper.

The companion paper proposes that an appreciation of evolutionary conflicts over IBI can help to explain the sleep of human infants [9]. This paper proposes that an appreciation of these conflicts may also help to understand some causes of secondary infertility (i.e. infertility after the birth of a child). Specifically, it is proposed that persistent cells of older offspring in mother’s bodies may interfere with the conception or implantation of subsequent embryos as an adaptation to extend IBIs.
